# Is the Risk to Develop Osteoradionecrosis of the Jaws Following IMRT for Head and Neck Cancer Related to Co-Factors?

**DOI:** 10.3390/medicina57050468

**Published:** 2021-05-11

**Authors:** Eli Rosenfeld, Bassel Eid, Daya Masri, Aron Popovtzer, Aviram Mizrachi, Gavriel Chaushu

**Affiliations:** 1 Department of Oral & Maxillofacial Surgery, Beilinson Hospital, Rabin Medical Center, Petah Tikva 4941492, Israel; dayama@clalit.org.il (D.M.); gavrielce@clalit.org.il (G.C.); 2 The Maurice and Gabriela Goldschleger School of Dental Medicine, Tel Aviv University, Tel Aviv 6997801, Israel; bassel227@gmail.com; 3 Sharett Institute of Oncology, Hadassah Medical Center, Jerusalem 9574401, Israel; aron@hadassah.org.il; 4 Department of Otorhinolaryngology—Head and Neck Surgery, Rabin Medical Center, Petah Tikva 4941492, Israel; AviramM2@clalit.org.il; 5 Sackler Faculty of Medicine, Tel Aviv University, Tel Aviv 6997801, Israel; 6 Department of Oral and Maxillofacial Surgery, The Maurice and Gabriela Goldschleger School of Dental Medicine, Tel Aviv University, Tel Aviv 6997801, Israel

**Keywords:** osteoradionecrosis of the jaw, osteo-radionecrosis (ORN), intensity modulated radiation therapy (IMRT)

## Abstract

*Background and Objectives:* Determine the contribution of coexisting factors to the risk to develop Osteoradionecrosis (ORN) of the jaws among patients who have received radiotherapy by intensity modulated radiation therapy (IMRT) for head and neck cancer (HNC) between 2013 and 2016, in a single medical center. *Materials and Methods:* The records of all patients treated with IMRT for HNC between 2013 and 2016 in The Davidoff Center for the treatment and Research of Cancer in Rabin Medical Center—Beilinson hospital, Petah-Tikva, Israel were screened. Patients who have received a minimum mean dose of 40 Gy to the oral cavity entered the research and their medical records were retrospectively reviewed. Collected background data included: age, gender, smoking, diabetes mellitus (DM), ASA score, mean and maximal radiation doses (Gy), and diseases characteristics including histological diagnosis, primary tumor site, and disease stage. *Results:* A total of 1232 patients were surveyed. Out of all screened patients, 93 received a minimum mean dose of 40 Gy to the oral cavity. Out of the 93 patients, 7 (7.52%) developed ORN (ORN+) and 86 did not develop ORN (ORN−). Tumor type in all seven patients in the ORN+ group was Squamous Cell Carcinoma (SCC). In three out of those seven patients (42.9%), the tumor was located in the mandible. *Conclusions:* within the limits of the relatively small cohort in the current study, we suggest that the development of ORN due to Radiation therapy (RT) with IMRT is related significantly only to the location of a tumor in the mandible. Other co-factors do not significantly increase the risk to develop ORN when RT is delivered via IMRT.

## 1. Introduction

First description of the phenomenon of necrosis of the jaws post radiation therapy later termed osteoradionecrosis (ORN) is attributed to Regaud in the early 1920s [1]. The definition of ORN in recent literature is the presence of exposed bone that does not undergo spontaneous healing for three months, and the lack of evidence of tumor recurrence [2].

The clinical findings and symptoms that accompany the exposure of necrotic bone include pain and recurrent infections of the exposed bone and pus secretion. The presence of exposed necrotic bone in the oral cavity can cause halitosis, impaired sense of taste and food impaction, depending on the location of the lesion, it might be associated also with symptoms of trismus and/or paresthesia. [3] 

**Figure 1:**
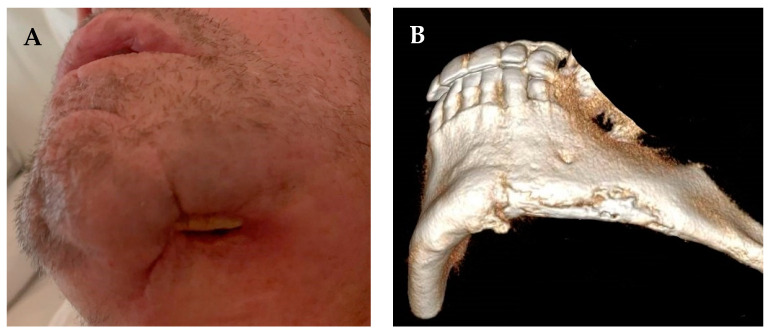
Mandibular ORN following mandibulotomy and radiotherapy for squamous cell carcinoma of the base of tongue. (**A**) clinical image demonstrating dehiscence of the soft tissue and exposure of necrotic bone on the inferior border of the left mandibular parasymphysis and body and (**B**) a 3-dimensional reconstruction of the CT demonstrating sequestration of the inferior mandibular border.

This phenomenon occurs almost exclusively in the mandible (Figure 1). In the minority of cases where it occurs in the maxilla, ORN progresses more slowly, and the extent of the bony damage is usually less severe. Differences between the jaws are probably related to the fact that the blood supply to the mandible is significantly reduced compared to the maxilla and that the mandible absorbs greater pressure and load compared to the maxilla [4].

The histopathology of ORN is characterized by a lack of osteoblasts, and replacement of normal bone marrow with fibrotic tissue. There is apparent absence of inflammatory cells, while a multitude number of bacteria (mostly anaerobic) and fungus can be detected in ORN specimens [5,6].

The reported prevalence of the phenomenon is variable, ranging between 0.4% and 56.0% [2,7]. With the implementation of Intensity Modulated Radiation Therapy (IMRT) in the treatment of head and neck cancers (HNCs), a significant decrease in the extent of ORN development can be seen, with newer reports ranging between 5% and 15%, mostly in patients older than 55 [2].

The risk factors for the development of ORN include local, systemic and also genetic factors [8,9,10,11]. Local factors include tumor size, xerostomia, neglected dental status, poor oral hygiene and associated local trauma, such as tooth extraction/surgery before or after radiotherapy. Systemic factors include immune system malfunction, malnutrition, peripheral vascular disease, heavy smoking, and excessive alcohol consumption. Generally, the risk of developing ORN is lower in edentulous patients. The dose of radiation is obviously also related to the development of ORN which is more prevalent in patients who have been exposed to 60–70 Gy during radiation therapy (RT) and have received chemotherapy in addition to RT.

The pathogenesis of ORN is not completely understood. In 1970 Meyer suggested the Radiation-induced Osteomyelitis theory [12], according to which, the radiation causes changes and cellular death in soft tissue leading to dehiscence and bone exposure, resulting in secondary infection that leads to ORN. This theory was refuted by Marx (1983) who proposed that ORN is caused by endarteritis secondary to the RT which results in tissue hypoxia, hypocellularity, and hypovascularity. Later, a breakdown of cellular and extracellular tissue causes a chronic nonhealing wound with metabolic demands that cannot be met due to the persistent hypoxia. This persistent hypoxia is the principal reason for the use of Hyperbaric Oxygen Therapy (HBOT) in the treatment of ORN [13]. However, the most widely accepted theory regarding the pathogenesis of ORN is the fibro-atrophic theory suggested by Delanian and Lefaix (2004) claiming that the radiation causes both direct endothelial cell injury and indirect injury through the generation of free radicals and reactive oxygen species (ROS). The oxidative stress is aggravated by further production of ROS by the acute inflammation that develops due to the endothelial injury, small vessel thrombosis, ischemia and necrosis. Cytokine production (fibroblast growth factor β, transforming growth factor β1, tumor necrosis factor α and interleukins) that accompanies each of these phases ultimately leads to “trans-differentiation” of fibroblasts into myofibroblasts. The ultimate result in the tissue is greater proliferation and production of abnormal extracellular matrix. The radiation also causes depletion of osteoblasts, osteocytes and osteoclasts eventually leading to the replacement of bony tissue with a fibrous matrix. The combination of reduced cellularity, reduced vascularity and fibrosis leaves brittle soft tissue with a poor recovery ability [14].

In accordance with the variety of definitions for the phenomenon, a number of classifications have been published in the literature [13,15,16,17] However, the most widely used classification of ORN today is the classification suggested by Notani et al. (2014) [17], based on the extent of the lesion: 

I—ORN confined to alveolar bone, II—ORN limited to the alveolar bone and/or mandible above the level of the inferior alveolar canal, III—ORN involving the mandible below the level of the inferior alveolar canal and/or skin fistula and/or pathological fracture. 

Treatment of ORN is primarily focused on prevention. Patients are instructed to undergo dental treatment and necessary extractions prior the radiotherapy in order to reduce the causes of infections and prevent the need for subsequent surgical treatments [18]. Post RT, patients undergo minimally invasive restorative dental treatments, frequent visits to the dental hygienists and ultra-conservative dental treatment in order to avoid extractions. 

Early lesions, mainly asymptomatic ones, may be treated conservatively by smoothing exposed bone margins to prevent mucosal irritation, chlorhexidine-based rinses to prevent secondary infection and periodic antibiotic courses [9]. HBOT treatment, intended to raise the oxygen pressure in the hypoxic tissue, encouraging angiogenesis, fibroblast proliferation and collagen synthesis [13,19]. Another conservative adjunct is medication treatment with Tocopherol and Pentoxifylline. Tocopherol, a powerful antioxidant that also functions as a co-enzyme that protects cells from free radicals formed as a result of RT. In addition, it inhibits TNFα and reduces the development of fibrosis. Pentoxifylline, a methylxanthine derivative, has an inhibitory effect on the activation of fibroblasts and raises collagenase activity [3]. However, in cases of advanced lesion which does not resolve under conservative treatment or in case of pathological fracture, extensive resection of the necrotic bone and reconstruction with a free flap usually is a definitive treatment for ORN. Extensive resection of the bone in combination with coverage of the area with a free flap and proper blood supply allows healing of the lesion [20].

The current study aimed to review the records of all patients treated with IMRT for HNC between 2013 and 2016 in a single medical center. Records of patients who have received a minimum mean dose of 40 Gy to the oral cavity were further evaluated. The development of ORN and for relationship of co-factors on the development of ORN was analyzed.

## 2. Materials and Methods

A cross sectional, retrospective and analytical study was conducted in the department of Oral and Maxillofacial surgery and The Davidoff Center for the treatment and Research of Cancer in Rabin Medical Center—Beilinson, Petah-Tikva, Israel. The medical records of all patients who underwent IMRT for HNC between the years 2013 and 2016 were screened.

Inclusion criteria: all patients who have received a mean dose of at least 40 Gy to the oral cavity between the years 2013 and 2016. 

Exclusion criteria: lack of data in the medical records, patients who have received a mean dose of less than 40 Gy to the oral cavity. 

Background data collection:

Medical records of all patients who met the inclusion criteria were thoroughly examined. Background data included: age, gender, ASA score [21], diabetes mellitus (DM), smoking status, mean and maximal radiation doses (Gy), and diseases characteristics including histological diagnosis, primary tumor site, and disease stage. The outcome parameter was the development of ORN.

### Statistical Analysis

Data were entered and analyzed in SPSS version 24. First, descriptive statistics were produced, while means and standard deviations were calculated for all continuous measures. All measures presented normal distribution (*p* > 0.05). Statistical analysis was performed using a *t*-test for continuous variables and Chi-square test for non-continuous variables. Significance was reported as *p* < 0.05.

## 3. Results

In the period between 2013 and 2016, 1232 patients were treated with IMRT for HNCs and benign conditions, indications for the RT are summarized in Table 1.

Out of the 1232 patients, 93 patients have received a mean dose of at least 40 Gy to the oral cavity and were enrolled in the study (study cohort). Within the study cohort of 93 patients, 7 patients (7.52%) developed ORN.

### 3.1. Gender and Age

Within the study cohort, 67 were males (72%), 26 were females (28%). Gender distribution within the ORN+ did not differ significantly from the distribution within the ORN− group (*p* = 0.36). 

The ages of the patients in the study cohort ranged between 16 and 93 years, with mean age of 61.95 years (SD 16.32). Within the ORN+ group the mean age was 70.57 years (SD 12.16), and within the ORN− group the mean age was 61.24 years (SD 16.46). The differences between the ages within the groups were not statistically significant (*p* = 0.73). All demographic data are summarized in Table 2.

### 3.2. Radiation Dose

The mean radiation dose to the oral cavity within the study cohort ranged from 40.3 Gy to 101.9 Gy, with mean value of 55.45 Gy (SD 16.5). Within the ORN+ group the mean radiation dose ranged between 42.9 Gy and 84 Gy, with a mean value of 54.71 (SD15.4), within the ORN− group the mean radiation dose ranged from 40.3 Gy to 101.9 Gy, with a mean value of 55.5 (SD 16.6). The differences of the mean radiation doses between the ORN+ and ORN− groups were not statistically significant (*p* = 0.45).

The maximal radiation dose to the oral cavity within the study cohort ranged from 69.8 Gy to 111.9 Gy (mean 100.78, SD 8). Within the ORN+ group the maximal radiation dose ranged between 90.6 Gy and 106.5 Gy, with a mean value of 100.6 Gy (SD 5.7), within the ORN− group, the mean radiation dose ranged from 69.8 Gy to 111.9 Gy, with a mean value of 108 Gy (SD 8.2). The differences of the mean radiation doses between the ORN+ and ORN− groups were not statistically significant (*p* = 0.42).

### 3.3. Cofactors and Tumor Characteristics

Cofactors that were analyzed in the study included smoking, diabetic status, and the ASA score. All the data of these factors are summarized in Table 3. Tumor-related factors included the pathological diagnosis, tumor site and tumor stage. All tumor characteristics are summarized in Table 4. Table 5 Summarizes all the data collected on the ORN+ group.

### 3.4. Smoking

Within the study cohort, 30 were active smokers (32.3%). Within the ORN+ group 2 patients (28.6%) were active smokers, compared to 28 patients (32.6%) in the ORN− group. These differences were not statistically significant (*p* = 0.83).

### 3.5. Diabetic State

A total of 17 patients within the study cohort had DM; in 9 of them the diabetic state was uncontrolled. None of the diabetic patients developed ORN. The difference between the ORN+ and ORN− groups regarding the diabetic status was not statistically significant (*p* = 0.43).

### 3.6. ASA Score

ASA score distribution within each group is presented in Table 3. The differences were not statistically significant (*p* = 0.48).

### 3.7. Tumor Characteristics 

The pathological diagnoses of patients within the study cohort included squamous cell carcinoma (SCC), salivary gland malignancies, osteogenic sarcoma, lymphoma, papillary thyroid carcinoma, paraganglioma and basal cell carcinoma. For the statistical analysis all the diagnoses other than SCC and SGM were pooled into a single group. The distribution of the diagnoses is presented in Table 4. A total of 81 out of 93 (87.1%) of the patients in the study cohort had SCC. Four patients (4.3%) had salivary gland tumor and the remaining eight patients (8.6%) had other malignancies. All seven patients in the ORN+ group had SCC, yet these differences were not statistically significant (*p* = 0.571)

Patients in all disease stages 1 to 4b were included in the study cohort. The distribution of disease stages of the patients in each group is presented in Table 4. No significant differences were found between the patients that did not develop ORN and patients that did (*p* = 0.633).

### 3.8. Tumor Site

Patients within the study cohort were irradiated for tumors in various locations within the head and neck region. The most common sites in this cohort were the nasopharynx (22.6%), oropharynx (19.4%), tongue (8.6%), buccal mucosa (7.5%), and the mandibular alveolar ridge (6.5%). The distribution of tumor sites between the ORN+ and the ORN− groups differed significantly (*p* = 0.006). Out of the seven patients of the ORN+ group, three had a mandibular alveolar ridge tumor, two patients had a nasopharyngeal tumor, one patient had a palatal tumor and one patient had an oropharyngeal tumor. The detailed distribution of sites in all groups is presented in Table 4.

## 4. Discussion

RT plays an important role in the treatment of malignancies of the head and neck; RT can be used in different indications as a definitive, complimentary or as a palliative treatment. One of the most problematic sequalae of RT in the head and neck is of ORN, a late radiation toxicity characterized by the ulceration of the soft tissue and the exposure of necrotic bone that does not undergo spontaneous healing. The jaws, opposed to other bones in the skeleton, face challenging conditions. In the oral cavity there is constant contamination, thin, soft tissue coverage, and exposure to irritation presented by the processing of various foods of different textures and temperatures. The presence of teeth that might necessitate surgical intervention, exposes the jaws and their surrounding soft tissues to injuries, further increasing the risk of bone injury. Following radiation therapy, the vulnerability of the tissues is increased due to the reduced cellularity, reduced vascularity and fibrosis weakening the soft tissue coverage with its impaired recovery ability. In the mandible the presence of thick cortical plates with lower turnover and the limited blood supply via the inferior alveolar artery further increase the risk of development of ORN.

In the mid-1990s, the concept of IMRT was introduced to the field of radiation oncology [22]. IMRT enabled delivery of 3D conformal RT with computer optimized inverse treatment planning together with intensity modulation of the radiation beam during the treatment [23]. In the field of HNC, the utilization of IMRT enabled to selective reduction in doses to healthy critical structures such as the parotid gland or the mandible even if they lie adjacent to or surrounded by tumor [24]. Indeed, this advent has lowered the reported incidence of ORN, De Felice et al. in their review of ORN and IMRT; presented reports of ORN incidences in the range of 0 to 6.2% [25]. 

In this retrospective study, we examined the medical records of 1232 patients who have received RT for HNCs. Out of all screened patients, 93 received a minimum mean dose of 40 Gy to the oral cavity. A total of 7 patients out of 93 (7.52%) developed ORN. Chronopoulos et al. [2] found that the most commonly reported rates of ORN development are between 5 and 15%. Our findings settle with this report and are somewhat higher compared to incidence rates found by De Felice [25].

### 4.1. Gender and Age

When looking into cofactors that could be related to the development of ORN, this study has shown some interesting findings. The mean age of the 93 patients who have received a mean dose of at least 40 Gy to the oral cavity was 61.95 years; however, no significant difference was found between the mean age of patients that developed ORN (70.57 years) and the patients that did not develop ORN (61.24 years). Similarly, gender was not found a significant factor in the development of ORN in this study. 

Sathasivam et al. as well as Renda et al. also looked into risk factors for the development of ORN [4,26]. Both papers did not find correlation between age or gender and increased risk for the development of ORN. Chronopoulos et al. [2] described a higher incidence in patients older than 55; gender differences were not addressed in his review. The cohort of Chronopoulos included both patients that were treated with IMRT as well as patients that were treated with external beam RT delivery systems. In the study by Sathasivam all patients were treated with external beam RT. In the cohort of Renda all patients were treated with IMRT. This means that the gender is probably not a significant factor in the risk to develop ORN regardless of the radiation method employed. Regarding age, our findings support the reports by Renda.

### 4.2. Comorbidities

In our study we searched for the contribution of comorbidities and preexisting conditions to the risk of developing ORN. Specifically, we looked for active smoking, DM, and the ASA score. In our cohort, none of these factors were found to contribute significantly to the risk of ORN development. Several studies have reported of significant correlation between smoking, and ORN development [4,17,27]. Indeed, active smoking was suggested as a contributing factor to the development of ORN, yet Owosho et al. [8], in a cohort of 1023 patients treated with IMRT in Memorial Sloan Kettering Cancer Center for oral cavity cancer and oropharyngeal cancer, did not find significant correlation between continued smoking and increased risk to develop ORN. 

Diabetes mellitus has previously linked to increased risk to develop ORN [4,26]. In this study no significant correlation was found between DM and development of ORN; in fact, none of the diabetic patients in the study cohort developed ORN. 

### 4.3. Tumor Characteristics

All patients who developed ORN in this study had SCC, yet as the vast majority of HNCs are SCCs, this finding is not statistically significant. Tumor stage as well was not found to be a significant factor in the development of SCC in this study. However, tumor site was found to be highly significant (*p* = 0.006).

Within the study cohort, six patients 6.5% had a tumor on the mandibular alveolar ridge, three of which developed ORN. This means that 50% of the patients with tumor of the mandible within the cohort developed ORN, these represent 42.9% of ORN+ patients compared to 3.5% of the patients in the ORN− group. This difference was found to be statistically highly significant (*p* = 0.006). 

The development of ORN is the result of significant changes within the irradiated bone as well as its soft tissue coverage [12,13,14]. Whether the main reason for the phenomenon lays in the resulting hypoxia, hypovascularity, and the hypocellularity as claimed by Marx [13], or in the fibroatrophic processes that occur due to the accumulation of free radicals and ROSs as claimed by Delanian [14], the result is a non-healing wound. In this study we looked for factors that would logically increase the risk to develop this condition such as radiation doses, physical status as reflected by ASA score [21] and DM. 

DM increases predisposition to develop microangiopathy and peripheral vascular disease. In an irradiated field, as previously discussed, blood supply is already interrupted due to endothelial cell injury and fibrosis of blood vessels. Hence, it would be logic to expect that the risk to develop ORN would be increased in diabetic patients. In our cohort, however, no correlation was found between DM and ORN. In fact, the only significant factor that was found in our study was the tumor site. A total of 9 out of 93 patients had uncontrolled DM yet none of which had mandibular tumor. Alcohol consumption was suggested in several articles as a risk factor for development of ORN [8]. In this study, however, alcohol consumption was not analyzed as we did not have the information regarding alcohol consumption habits for the whole population.

With the advent of IMRT in treatment of HNCs, the severity and incidence of radiation associated morbidities improved significantly. This improvement is the result of minimizing radiation exposure of healthy tissues [8,22,23,28]. As previously discussed, rates of ORN are lower in reports since the dawn of IMRT era. When reviewing the reports in the literature for risk factors to the development of ORN, the issue of irradiation method must be addressed. The presence of reports that are based on data from the “pre IMRT era” as well as reports that include a non-homogenous population of patients with regard to the method of irradiation creates a bias. With the decline in the incidence of ORN, the significance of attributing factors has also declined. In his report of one of the largest cohorts of HNC, Owosho et al., collected data regarding smoking as well as DM, yet both these factors were not reported as significant risk factors [8]. Though our cohort is smaller than Owosho’s cohort of The Memorial Sloan Kettering Cancer Center, many of our findings settle with this report. The fact that, apart from tumor site, none of the cofactors and preexisting conditions that were inspected in this study were not found to be statistically significant could actually be a noteworthy outcome. Obviously smoking and DM do impair wound healing yet, in the IMRT era, the possibility to spare healthy tissues dramatically lowered the importance of such cofactors. This might explain why, even in his large cohort, Owosho could not find that smoking and DM significantly increased the risk to develop ORN.

## 5. Conclusions

ORN is a serious complication of RT for HNCs. While the development of ORN is usually attributed to various patient related and tumor related factors, such as diabetes, smoking, and the stage of the disease, the introduction of IMRT could have minimized the significance of these influences. Within the limits of the relatively small cohort in the current study, these preliminary results suggest that, following IMRT irradiation for the head and neck, the risk to develop ORN is mainly related to the tumor site while other health-related and tumor-related factors play by far a smaller role. Further study on a large cohort of patients that were treated with IMRT is necessary to validate these findings.

## Figures and Tables

**Table 1:** Indications for the RT.

Indications for the RT	No. of Patients
Benign neoplasm of middle ear, nasal cavity and accessory sinuses	8
Benign neoplasm of cerebral meninges	24
Neoplasm of uncertain or unknown behavior of larynx	57
Malignant neoplasm of oropharynx, unspecified	16
Benign neoplasm of parotid gland	20
Malignant neoplasm of parotid gland	44
Malignant neoplasm of anterior floor of mouth	29
Malignant neoplasm of skin of scalp and neck	130
Malignant neoplasm of skin of other and unspecified parts of face	93
Malignant neoplasm of skin of ear and external auricular canal	27
Malignant neoplasm of nasal cavity	41
Overlapping malignant neoplasm of lip, oral cavity and pharynx	8
Malignant neoplasm of glottis	160
Malignant neoplasm of brain, unspecified	262
Secondary malignant neoplasm of brain and cerebral meninges	91
Malignant neoplasm of thyroid gland	83
Malignant neoplasm of cerebral meninges	24
Malignant neoplasm of maxillary sinus	29
Malignant neoplasm of tongue, unspecified	79
Malignant neoplasm of carotid body	2
Malignant neoplasm of parathyroid gland	1
Malignant neoplasm of cranio-pharyngeal duct	1
Malignant neoplasm of pineal gland	3
	**Total: 1232**

**Table 2:** Demographic data.

Co-Factor	ORN−	ORN+	Study Cohort (93)	*p* Value
Male	63 (73.3%)	4 (57.1%)	67 (72%)	0.361
Female	23 (26.7%)	3 (42.9%)	26 (28%)	
Age (mean (years) ± SD)	61.24 ± 16.46	70.57 ± 12.16	61.95 ± 16.32	
Age Range (years)	16–93	57–89	16–93	

**Table 3:** Cofactors.

Co-Factor		ORN−	ORN+	Study Cohort (*N* = 93)	*p* Value
**Smoking**	No	58 (67.4%)	5 (71.4%)	63 (67.7%)	0.828
	Yes	28 (32.6%)	2 (28.6%)	30 (32.3%)	
**DM**	No	69 (80.2%)	7 (100%)	76 (81.7%)	0.429
	Controlled	8 (9.3%)	0	8 (8.6%)	
	Uncontrolled	9 (10.5%)	0	9 (9.7%)	
**ASA score**	1	16 (18.6%)	0	16 (17.2%)	0.482
	2	35 (40.7%)	3 (42.8%)	38 (40.9%)	
	3	24 (27.9%)	2 (28.6%)	26 (28.0%)	
	4	11 (12.8%)	2 (28.6%)	13 (14.0%)	

**Table 4:** Tumor characteristics.

Characteristic		ORN−	ORN+	Study Cohort (*N* = 93)	*p*-Value
**Pathological diagnosis**	SCC	74 (86.0%)	7 (100%)	81 (87.1%)	0.571
	Salivary gland	4 (4.7%)	0	4 (4.3%)	
	Other	8 (9.3%)	0	8 (8.6%)	
**Disease stage**	1	7 (8.1%)	0	7 (7.5%)	0.633
	2	6 (7.0%)	1 (14.3%)	7 (7.5%)	
	3	19 (22.1%)	2 (28.6%)	21 (22.6%)	
	4a	39 (45.3%)	4 (57.1%)	43 (46.3%)	
	4b	15 (17.5%)	0	15 (16.1%)	
**Tumor site**	Tongue	8 (9.3%)	0	8 (8.6%)	0.006
	Floor of mouth	5 (5.8%)	0	5 (5.4%)	
	Maxillary alv. Ridge	2 (2.3%)	0	2 (2.1%)	
	Mandibular alv. Ridge	3 (3.5%)	3 (42.8%)	6 (6.5%)	
	Retromolar trigone	1 (1.2%)	0	1 (1.1%)	
	Buccal mucosa	7 (8.1%)	0	7 (7.5%)	
	Oropharynx	17 (19.8%)	1 (14.3%)	18 (19.4%)	
	Nasopharynx	19 (22.1%)	2 (28.6%)	21 (22.6%)	
	Parotid	3 (3.5%)	0	3 (3.2%)	
	Palate	1 (1.2%)	1 (14.3%)	2 (2.1%)	
	Other	20 (23.3%)	0	20 (21.5%)	

**Table 5:** ORN+ patients characteristics.

Patient No’	Gender	Age	ASA Score	DM	Smoking	Tumor Type	Tumor Stage	Tumor Site	Oral Cavity MAX (Gy)	Oral Cavity MEAN (Gy)
**1**	F	73	3	NO	NO	SCC	4a	Mand. Alv. Ridge	99.1	53.4
**2**	F	89	4	NO	NO	SCC	3	Mand. Alv. Ridge	104.7	47.7
**3**	M	59	2	NO	NO	SCC	3	Oropharynx	97.4	44.5
**4**	M	57	4	NO	YES	SCC	4a	Palate	105.8	84.0
**5**	F	77	3	NO	NO	SCC	4a	Mand. Alv. Ridge	106.5	66.9
**6**	M	79	2	NO	YES	SCC	4a	Nasopharynx	90.6	42.9
**7**	M	60	2	NO	NO	SCC	2	Nasopharynx	100.1	43.6

## Data Availability

Data available on request due to restrictions of the ethical committee approval.
